# Clustering of cognate proteins among distinct proteomes derived from multiple links to a single seed sequence

**DOI:** 10.1186/1471-2105-9-141

**Published:** 2008-03-05

**Authors:** Adriano Barbosa-Silva, Venkata P Satagopam, Reinhard Schneider, J Miguel Ortega

**Affiliations:** 1 Laboratório de Biodados, Dep. Bioquímica e Imunologia, Instituto de Ciências Biológicas, UFMG, Av. Antônio Carlos 6627, Belo Horizonte, MG, Brasil; 2 European Molecular Biology Laboratory, EMBL-Heidelberg, Meyerhofstr 69117, Heidelberg, Germany

## Abstract

**Background:**

Modern proteomes evolved by modification of pre-existing ones. It is extremely important to comparative biology that related proteins be identified as members of the same cognate group, since a characterized putative homolog could be used to find clues about the function of uncharacterized proteins from the same group. Typically, databases of related proteins focus on those from completely-sequenced genomes. Unfortunately, relatively few organisms have had their genomes fully sequenced; accordingly, many proteins are ignored by the currently available databases of cognate proteins, despite the high amount of important genes that are functionally described only for these incomplete proteomes.

**Results:**

We have developed a method to cluster cognate proteins from multiple organisms beginning with only one sequence, through connectivity saturation with that Seed sequence. We show that the generated clusters are in agreement with some other approaches based on full genome comparison.

**Conclusion:**

The method produced results that are as reliable as those produced by conventional clustering approaches. Generating clusters based only on individual proteins of interest is less time consuming than generating clusters for whole proteomes.

## Background

Modern proteomes are generated from ancestral ones by modifications that occur at the DNA level of the corresponding coding genomes. Such modifications (termed genetic variations) have different sources, among them: mutations, genetic recombination and alternative splicing (the last occurring at the RNA level). All generate variability in the protein repository present in one population. As a result, after isolation and speciation events, populations carrying closely-related proteomes can produce highly related protein sets.

However, a great part of the cognate proteins encoded by distinct proteomes is strictly similar, at the sequence level, to their counterparts in related species. This similarity is more than structural, often reflecting also in the function of these proteins in the biological system. Proteins derived from a common ancestor are termed homologs.

There are different subtypes of homology relationships attributed to proteins based on an evolutionary point of view. Among these, we highlight those proposed by Sonnhammer and Koonin [1] which defines orthologs, in-paralogs and out-paralogs as subtypes of homolog protein/genes, which can be operationally used by bioinformatics tools.

Several approaches have been designed to cluster related sequences from different organisms into the same ortholog group. Some of them use either all-versus-all alignments among different species [2] or pair-wise alignment among target organisms [3] as well. Each of these techniques is based on information deposited for fully sequenced genomes, and generate distinct ortholog groups using customized algorithms or thresholds in their searches [4-6].

Despite the prosperity of methods for definition of ortholog proteins among complete proteomes, there are few developments when it is desired to define such groups when poorly sequenced or unfinished proteomes are included in the search. Furthermore, most of the available methods are used in a high-throughput way, considering the whole protein dataset.

Here, we propose a methodology for finding highly-related groups of proteins to one single desired Seed sequence, classifying each of them as potential orthologs or in-paralogs into complete and or unfinished proteomes.

## Methods

### Algorithm

#### Input dataset

Seed Linkage runs using as input a fasta formatted database and a MySQL [7] table that contains taxonomic information (obtained from NCBI Taxonomy database) for all sequences contained in the database. The fasta file is expected to contain seed sequences and all candidate sequences with which one aims to establish a clustering relationship. Using the access to the MySQL database the program automatically recognizes which sequence belongs to which organism, a procedure that facilitates the setup and limits BLAST searches to the set of interest (the ongoing hits). We have tested and established the best thresholds that allows a sequence to be selected as a correct member of a group, which allows the possibility of working with sequences from organisms with incompletely-sequenced genomes.

#### Alignment details

Alignments between sequences are obtained with NCBI BLAST: BLASTp program [8], 10^-10 ^E-value cutoff, low complexity filter off (-F f), tabular output (-m 8). BLAST parameters specified in the Seed Linkage configuration file can be altered by the user. To minimize the problem of fused genes/domains we defined 50% as the cut-off for both minimum identity and alignment coverage (with the Seed sequence), as explained by Remm *et al*. [3]; this too can be customized.

The main trait that distinguishes the Seed Linkage method from other approaches is the manner by which the alignments are conducted. The seed protein from the Seed Organism is used in a BLAST search against the full database, and the best hit from each organism is used as secondary query sequence. The secondary query is considered a bidirectional best hit subject (BBHsj) for its organism when the original Seed is its best hit from the Seed Organism. The BBHsj is considered as a putative ortholog in that organism; from the information contained in these alignments inparalogs are gathered as described below.

#### Inparalog search into Seed Organism

**Figure 1:**
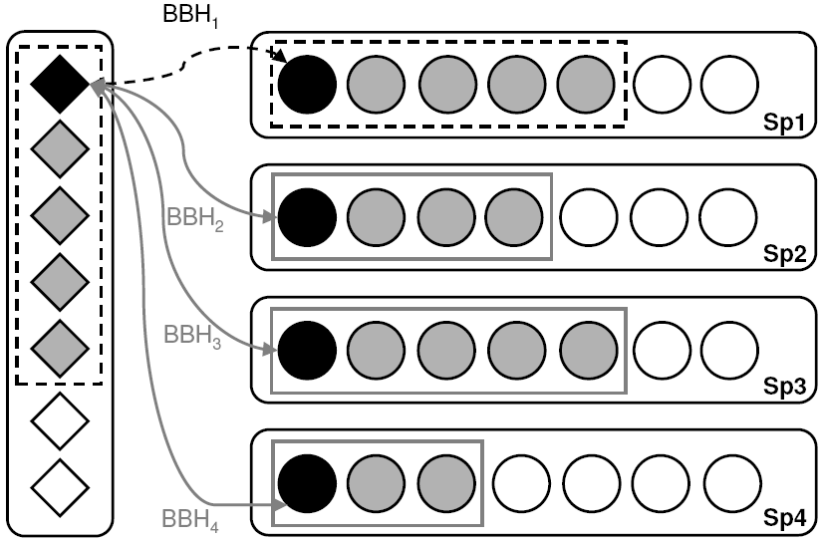
**BBH algorithm adopted for Seed Linkage**. The algorithm starts by aligning the Seed sequence from the Seed Organism (black diamond) to sequences from all other organisms in the database (circles in Candidate Species), searching for a BBH for each Species. The score BBH_1 _between the Seed and the highest-scoring sequence (black circle in Sp1) defines the *inparalog retrieval score limit *in Seed Organism. The inparalogs for the Seed Organism are those sequences whose alignment score between the Seed and the potential inparalog (grey diamonds) exceeds BBH_1 _(dashed boxes within Seed Organism). The BBH scores (BBH_1–4_) are used to filter potential inparalogs (grey circles) from the respective Candidate Species (Sp1-4, respectively) when the BBHsj from each species (black circles) are used as secondary queries against proteins from the Candidate Species genome. These thresholds aim to avoid the inclusion of additional spurious sequences in clusters (white diamonds and circles). Inclusion requires a BBH relationship between candidates (grey symbols to be incorporated) and already grouped sequences (black and grey symbols) within the respective Candidate Species.

The Seed-to-BBHsj score from the best scoring organism is used as a threshold to limit inparalog inclusion for proteins encoded by the Seed Organism. This means that only inparalog candidates that are more (or equally) similar to the seed sequence than the highest scoring BBHsj will be grouped with the Seed (Figure 1). Besides surpassing the thresholds, an inparalog candidate is grouped only if it shows a BBH relationship with either the Seed or with an already-grouped inparalog.

If the Seed does not establish any BBH relationship with sequences from other organisms, the *inparalog retrieval score limit *in the Seed Organism is set to a minimum value for the parameter called SEED-Inparalog_relative_score _given by the formula:

(I) SEED-Inparalog_relative_score _= Score_inparalog_vs_SEED/Score_SEED_vs_SEED

This parameter is set to a default value of 0.3 (see "Results") but can be customized during setup. This means that all inparalog candidates have to present a score that is at least 30% of the score of the Seed against itself. Similarly, for other searches, an inparalog candidate is grouped only if it shows a BBH relationship with either the Seed or with an already grouped inparalog (Figure 1, black or upstream grey diamonds).

#### Inparalog search for Candidate Species

Candidate Species are organisms where a BBHsj was found by the initial alignment to the Seed. The score of the BBHsj against the Seed is used to limit the inparalog search within this respective species, as shown in Figure 1. Moreover, an inparalog candidate is grouped only if it shows a BBH relationship with either the BBHsj or with an already grouped inparalog.

#### Iteration

All inparalogs from the Seed Organism, but not the grouped proteins from the Candidate Species (BBHsj and its inparalogs), are brought to the condition of Seed and the process of clustering is repeated either until it converges or by a limited number of rounds, set by the parameter "r" in the script. The default is r = 10, but this can also be customized during input. That means that all inparalogs from the Seed Organism are allowed to gather additional inparalogs, plus orthologs and their respective inparalogs in Candidate Species, until at the most the 10^th ^search for inparalogs from the Seed Organism is done. However, iterations will always respect the *inparalog retrieval score limit *defined by the original Seed in the Seed Organism; and the score between the added inparalog used as Seed against its BBHsj as threshold to limit the inparalog inclusion from the Candidate Species.

#### Search of almost identical inparalog candidates

**Figure 2:**
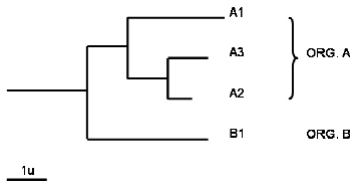
**Distance tree showing very similar inparalogs A2 and A3**. The best hit of sequence A3 (Seed), within organism B, is B1. However, when B1 is reciprocally aligned against sequences from organism A, A2 is the best hit, not A3, since A2 and A3 are very similar inparalogs. B1 is considered to form a BBH with A3 (Seed) if A3 appears at the 2^nd ^position in the reciprocal alignment and A2 has a BBH relationship with A3. Another 'hidden inparalogy' case occurs with inparalog searches beginning with A1 being A2 and A3 inparalog candidates. A2 is considered to form a BBH with A1 (Seed) if A1 appears at the 2^nd ^position in the reciprocal alignment and A2 has a BBH relationship with A3.

The tree diagram in Figure 2 exemplifies a case of what we term 'hidden inparalogy'. Consider sequence A1 from organism A (as either Seed or BBHsj), and two other sequences A2 and A3 generated by a lineage-specific duplication event so they are more similar to each other than to sequence A1. Then, A2 and A3 will not match A1 reciprocally; instead, they will match each other. To solve this problem, when an already-grouped protein (black and grey symbols in Figure 1, e.g. A1) is 2^nd ^in the returned alignment, the candidate is considered to have a reciprocal match, given that the pair A2-A3 does establish a BBH.

#### Search initiated by a Seed with an almost identical inparalog

Similar to the previous case, when a sequence A3 from organism A is used as Seed in a search for BBHsj from organism B, often sequence B1 matches reciprocally A3 in the organism A. However, consider sequence A2, an inparalog of A3, being more ancient in Organism A than A3. In this scenario, B1 will match reciprocally A2 instead of A3. To cope with this event, when Seed (e.g. A3) is 2^nd ^in the returned alignment, the candidate is considered to have a reciprocal match, given that the pair A2-A3 actually establishes a BBH.

#### Batch search

Though not aiming to generate clusters starting with multiple seed sequences, the Seed Linkage approach permits users to create such clusters by initially defining individual clusters through a batch search executed by the program, followed by the application of disambiguation rules. Thus, the user does not need to verify whether or not Seed sequences may be inparalogs.

#### Cluster disambiguation

Considering two clusters *i *and *j*, with size (number of sequences) N*i *and N*j*, we defined four relationships between clusters *i *and *j*:

(1) *i *is contained in *j*: in this case, if all the sequences of cluster *i *also belongs to cluster *j*, then cluster *i *is deleted.

(2) *i *is identical to cluster *j*: in this case, just one of the clusters is maintained.

(3) *i *is completely distinct from *j*: in this case both clusters are kept separately.

(4) *i *has elements in common with cluster *j*: in this case, if cluster *j *is larger than cluster *i*, and more than 50% of the sequences in cluster *i *are present in cluster *j*, than clusters *i *and *j *are merged, otherwise they are maintained separately.

### Implementation

#### Core scripts

The algorithm described above was implemented as a Linux command line script written in PHP command line interface [9]. The script is connected to a MySQL database where the information regarding to the sequence source database is stored as simple tables. Furthermore, it is also necessary to set a fasta sequence database and the path of the alignment software must be edited in the script. To facilitate database formatting we have developed a configuration file, in which the parameters pertaining to the script can be easily adjusted.

The main package consists of three files, clearly documented. Additional scripts to parse the model databases are provided within the package as Additional file 1.

### Algorithm evaluation

#### Manually curated database and non-related sequences

To validate the Seed Linkage approach a manually-curated dataset of 1363 trans-membrane proteins [10] previously grouped into 221 reference clusters was used as Seed against the proteome sequences of the following organisms: *C. elegans *(Cel), *D. melanogaster *(Dme) and *H. sapiens *(Hsa), comprising 19099, 14100 and 35118 sequences, respectively. The rebuilt clusters (RCs) were disambiguated and the resultant clusters were compared to the original reference clusters.

## Results

To develop and verify a procedure that results in clusters of proteins linked to seed sequences, we have chosen to assay the same manually-curated database of trans-membrane proteins that was used to verify clusters using the Inparanoid procedure. Briefly, Inparanoid drives automatic clustering of orthologs and inparalogs shared by different organisms with completely-sequenced genomes [3,11] or even by multiple proteomes from large taxonomic groups [4]. The manually-curated database contains 1363 sequences that are expected to constitute 221 manual clusters (MCs). The Seed Linkage procedure was applied to each sequence as Seed. As a challenge to correctly form clusters without including additional sequences, the BLAST database also included the complete proteomes from worm, fly and man, adding up to 66,954 entries. Grouped inparalog candidates were then iteratively treated as new Seeds and the resultant clusters were disambiguated.

### Clustering

**Figure 3:**
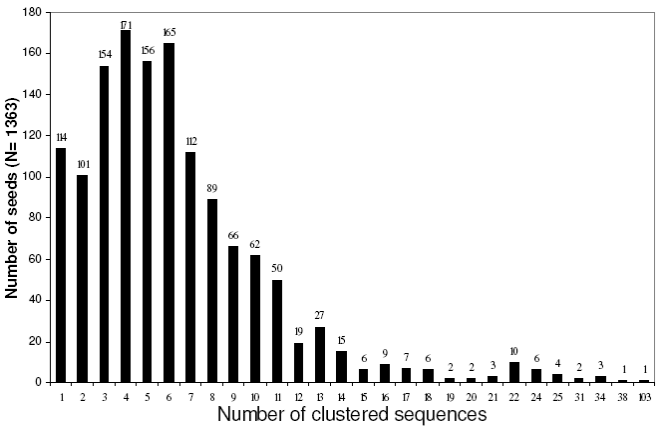
Distribution of number of sequences clustered by Seeds.

**Figure 4:**
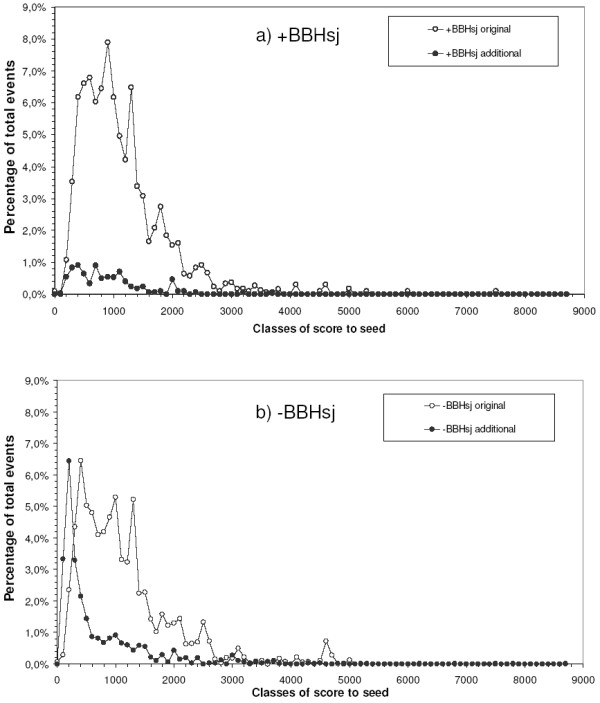
**Raw score distribution in clusters with (a) or lacking (b) a BBHsj reference sequence**. The alignment score to Seed for each gathering event was saved and the percentage of events within each class of score interval (binned in 50-bit increments) was determined. Legend: '+BBHsj original' and '+BBHsj additional', gathering events involving sequences respectively present or absent in manual clusters, in clusters with BBHsj reference sequence; '-BBHsj original' and '-BBHsj additional', gathering events involving sequences respectively present or absent in manual clusters, in clusters lacking a BBHsj reference sequence.

**Figure 5:**
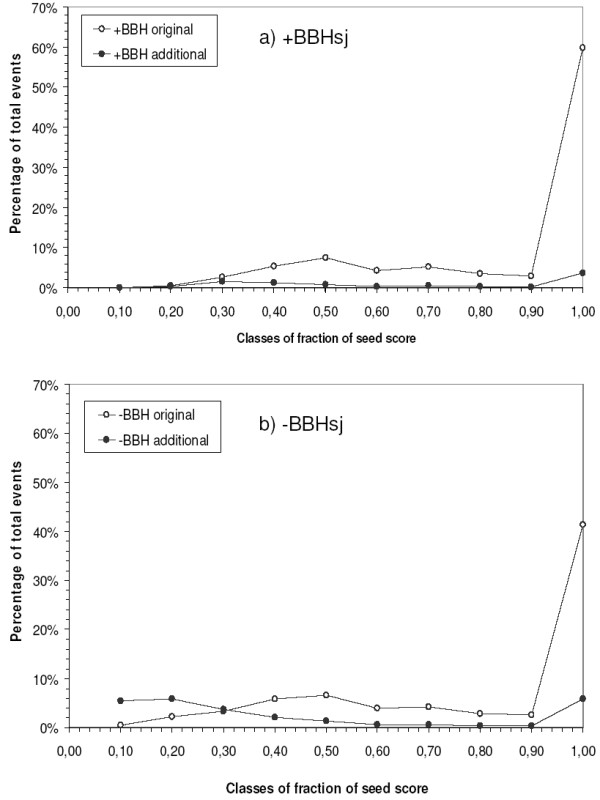
**Relative score distribution in clusters with (a) or lacking (b) a BBHsj sequence**. The alignment score to Seed divided by the score of Seed aligned to itself for each gathering event was saved and the percentage of events within each class of fraction of seed score interval (binned in .1 intervals) was determined. Legend: '+BBHsj original' and '+BBHsj additional', gathering events involving sequences respectively present or absent in manual clusters, in clusters with BBHsj reference sequence; '-BBHsj original' and '-BBHsj additional', gathering events involving sequences respectively present or absent in manual clusters, in clusters lacking a BBHsj reference sequence.

**Figure 6:**
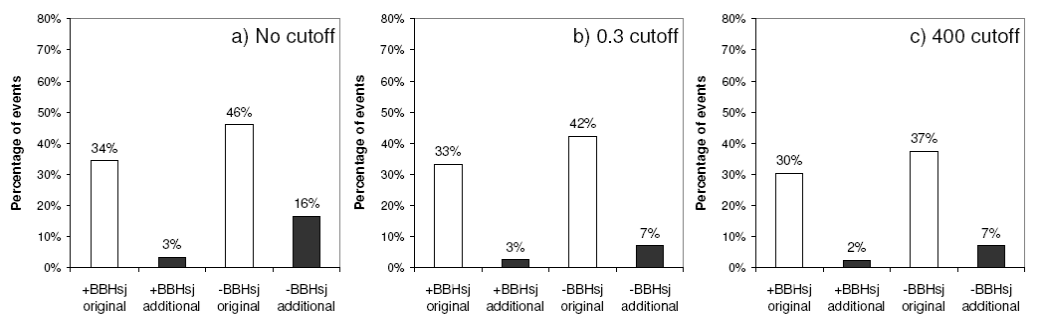
**Effects of relative and raw score in the percentage of sequences included into rebuild clusters**. Relative score of 0.3 (b) or raw score cutoff of 400 bits (c) were applied. Legend: '+BBHsj', clusters with BBHsj reference sequence;'-BBHsj', clusters lacking a BBHsj reference sequence.

**Figure 7:**
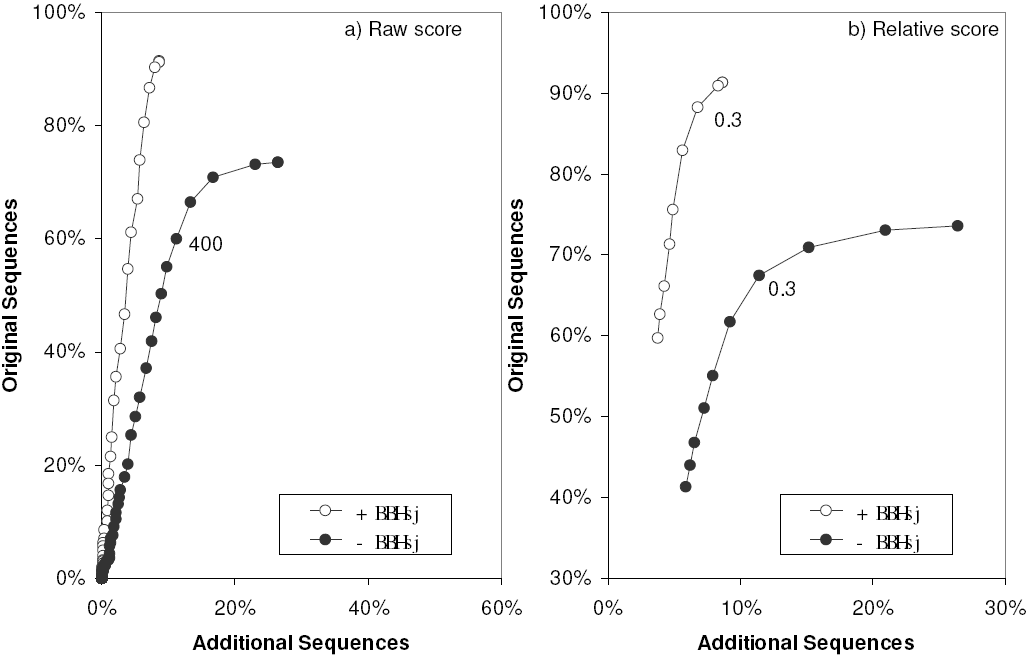
**ROC curves comparing the raw and relative score approach**. Each point represents cumulative recruitment over a given cutoff – highest percentages correspond to no cutoff; 400 raw score and 0.3 relative score are indicated.

**Figure 8:**
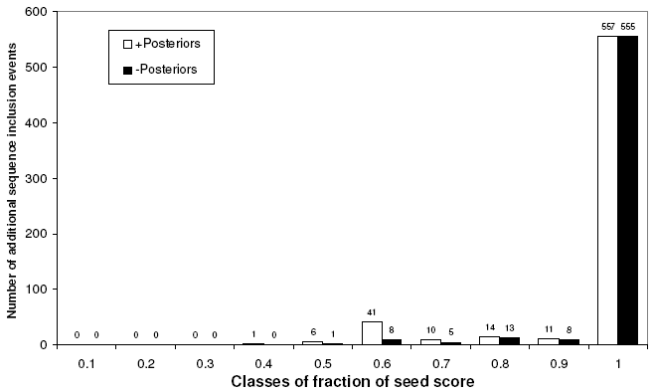
**Additional sequences recruited before original ones**. Additional sequences are shown per relative score range with (white bars) or without (black bars) original sequences being recruited subsequently.

The batch search script provided by the Seed Linkage package was used to run the 1363 individual processes. As detailed in "Methods," only the cutoff for the parameter SEED-Inparalog_relative_score _was omitted. Moreover, for the primary analysis (Figures 3, 4, 5, 6, 7, 8), cluster disambiguation was not performed. Of the generated clusters, 114 Seeds (8.36%, belonging to 17 curated clusters) remained as clusters of size 1 (actually not forming clusters), while the remaining 1249 sequences formed clusters whose size ranged from 2 up to 103 sequences (Figure 3). A lesser number of Seeds (173 sequences, 12.6%) participated in the largest clusters (>11 members). Sequences grouped by Seed Linkage always require a BBH relationship with either a Seed or previously grouped sequences so, in this experiment, a total of 7289 BBH events occurred, with 5582 events (76.6%) composed of sequences from the original trans-membrane dataset, and 1707 events involving additional sequences (737 distinct sequences). Thus, additional filtering seemed to be necessary to reduce the chance of mis-inclusion of spurious sequences; some of these might be out-paralogs, which could have diverged significantly to acquire new functionalities [1].

### Inparalog threshold evaluation

The thresholds (similarity = 50, alignment coverage = 50 and E-value < 10^-10^) used in BLAST alignments, together with the requirement of a BBH relationship for the sequence to be grouped, were not enough to limit the inclusion of additional sequences. In clusters lacking a reference BBHsj, this could be even more relevant since the search for inparalogs from the Seed Organism might tend to include outparalogs, which iteratively could include undesirable sequences. We decided to investigate two variables potentially useful in this task: raw and relative scores.

#### Raw score

We graphed the number of sequences from the original (trans-membrane) and additional (complementary proteomes) datasets that were recruited into the RCs against the raw score between each accepted inparalog candidate and either Seed (in Seed Organism) or BBHsj (in Candidate Species). Due to the expected possibility that the same sequence is gathered by different query sequences into distinct clusters, we reported all inclusion events for both original and additional sequences. The analysis was applied to clusters initiated either with or without a BBHsj reference hit (+BBHsj, -BBHsj). Figure 4 shows the raw score distribution in the batch search. For clusters initiated with a BBHsj (Figure 4a), the distribution of raw score resulting in the recruitment of additional sequences (filled symbols) resembles, although in lower proportion, that of original sequences (open symbols). This suggests that additional filtering would not significantly avoid gathering undesirable proteins. In these cases, the score Seed/BBHsj might have acted as a natural *inparalog retrieval score limit *for inparalog inclusion. However, the distribution of raw scores presented during the recruitment of additional sequences in the absence of a BBHsj (Figure 4b, filled symbols) was remarkably concentrated at the low raw scores, indicating that an *inparalog retrieval score limit *could be set to a value such as 400 bits, since 58% of the gathering events for additional sequences were concentrated below this range, as opposed to 19% for original sequences.

#### Relative score

The second analyzed parameter was the relative score. The ratio between the score of each gathering event and the score of Seed self-alignment was recorded. Again, in the presence of a BBHsj, the distribution of relative scores for the additional sequences is not distinct from those for the original ones (Figure 5a). However, Figure 5 shows that 57% of the gathering events of additional sequences occur in relative score ranges less than or equal to 0.3 (Figure 5b, filled symbols). In the same range, only 8% of gathering events of original sequences happen.

#### Alternative inparalogs thresholds definition

Considering the results above, we tested raw score 400 and relative score 0.3 as thresholds to minimize the inclusion of additional sequences to the MCs during Seed Linkage rebuilding. Data in Figure 6a shows that gathering events of original sequences represent 34% and 46% of the total alignments in clusters with and without a BBHsj, while gathering events of additional sequences represent 3% and 16%, respectively.

Adopting a threshold for inclusion of sequences with relative score higher than 0.3 (Figure 6b), the percentage of gathering events of original sequences decreased very little (to 33% and 42%, in clusters with and lacking a BBHsj, respectively). Similar behavior was observed for gathering events of additional sequences in clusters with a BBHsj (maintained at 3%). Remarkably, these events decreased to 7% in clusters without a BBHsj. Analyzing the effect of a raw score cutoff of 400 bits (Figure 6c), it is observed that the proportion of gathering events of original sequences decreased slightly more than using the relative score cutoff (Figure 6b), reaching 37% in the clusters lacking a BBHsj, without an effect on the gathering events of additional sequences in absence of BBHsj. Thus, adoption of a cutoff based on relative score 0.3 appears to be more efficient than using a raw scrore 400 cutoff. Furthermore, the adoption of an extra *inparalog retrieval score limit *does not appear to be necessary in when a BBHsj is established. A direct comparison between the two approaches – raw and relative score – is presented by ROC curves shown in Figure 7. In the presence of a BBHsj (open symbols) the percentage of accumulated original sequences increases linearly as the cutoff is made less stringent. However, for the clustering initiated without a BBHsj (solid symbols) a cutoff less than either 400 (Figure 7a) or 0.3 (Figure 7b) drives the procedure to recruit relatively more additional sequences than original sequences. The use of the relative cutoff appears to be advantageous, so it has been adopted as default.

### Post-filtering analysis of remaining additional sequences

Having applied the 0.3 relative score cutoff to clusters initiated both with and without a BBHsj, an analysis of the order in which the sequences were gathered reveals a curious phenomenon: the inclusion of original sequences after the inclusion of additional ones. Data in Figure 8 indicates that this scenario is frequent ("+Posteriors"), 52% of all events. These events comprise a total of 275 additional sequences, whereas 149 out of them (54.2%) have been gathered before an original one at least once. This might suggest that some gathered additional sequences were not recruited inappropriately. In fact, an analysis of the structural presence of trans-membrane domains (TM) shows that most of the additional sequences gathered with manual sequences *a posteriori *display at least two TM segments (133 out of 149, 89.3%). From the 126 additional sequences gathered at the last position by the algorithm, an additional 20 sequences also displayed at least two TM segments. Thus, possibly 133 plus 20 out of the 275 additional sequences (55.6%) might have had been suitably gathered.

### Cluster disambiguation and comparison to manually curated dataset

After applying the 0.3 threshold to filter all inparalog sequences below or equal to this level in clusters initiated without a BBHsj, the resultant clusters were disambiguated using the rules described in "Methods." After disambiguation, a total of 1638 unique sequences remained in the dataset. This represents an increase of 20% over the initial number of Seeds used, corresponding to the inclusion of 275 additional sequences to the original 1363 ones. As suggested above, some of them (153) might have not been inappropriately gathered, what would represent an increase of just 9.3% in the initial universe of 1363 original sequences.

With disambiguation, the initial 1249 clusters formed by the 1363 seed sequences were reduced to 263 clusters and 38 singlets (which belong to 19 MCs), which approximates the expected number of 221 MCs. Some MCs (59 out of 221, 26.7%) were split into more than one RC, while the remaining 162 MCs (73.3%) were represented by only one RC each. Conversely, 248 out of the 263 RCs (94.3%) were built with sequences from only a single MC, and within these, just 118 RCs (47.5% of 248) have gathered additional sequences. Indeed, in 100 of these 118 RCs, all additional sequences display more than two TM segments.

**Figure 9:**
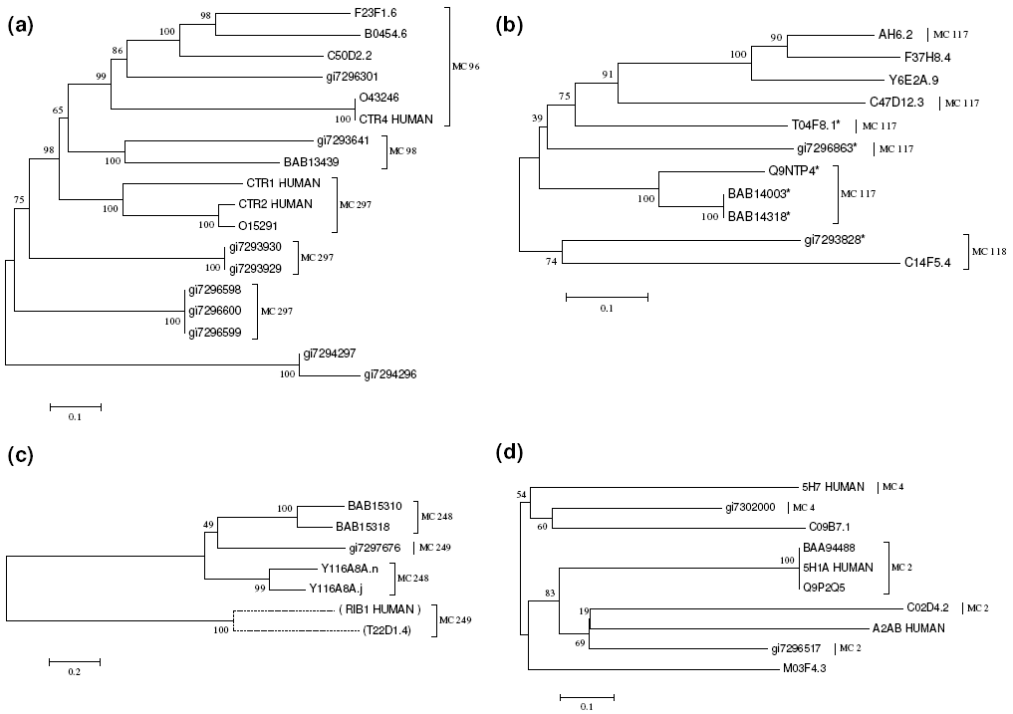
**Neighbor joining trees of merged clusters**. **(a) **Rebuilt Cluster RC1219 merges completely the three manually curated clusters MC96, MC98 and MC297 (represented in brackets) and additional sequences. **(b) **RC524 merges partially the clusters MC117 and MC118 and the remaining sequence from MC118 (C14F5.4, represented in the tree as the dashed branch), forms a singlet when used as Seed. When used as Seed, sequence gi72933828 gathers five sequences from MC117 (assigned by asterisks). **(c) **RC 1171 merges all sequences from MC248 with one sequence from MC 249. It is also represented the cluster RC1176, rebuilt by the remaining two sequences from MC249 (dashed branches). **(d) **RC12 gathers sequences from MC2 and MC4 have been merged. However, the remaining sequence from MC 2 (gi7296517) has formed the RC11 together with two additional sequences (not associated to the MC brackets).

Seed Linkage has merged sequences from 25 MCs to produce 15 of the 263 disambiguated RCs. This is unlikely to be explained by mixing of unrelated proteins since all alignments are required to surpass a 10^-10 ^E-value cutoff. From these 15 RCs, 12 result from the merging of all elements of the involved MCs. As an example, the neighbor joining (NJ) tree for the sequences present in RC1219 is shown in Figure 9a, wherein three MCs plus two additional sequences have been grouped. Moreover, the three remaining merged clusters represent exclusive cases also illustrated in Figure 9: RC254 has mixed all sequences from MC117 (size 9) together with one sequence from MC118 (size 2), the other sequence from MC118 has not built a cluster when used as Seed (represented on Figure 9b as a dashed branch). Note that, when used as Seed, sequence gi7293823 (MC118) was able to gather 5 out of 7 sequences from MC118 (labeled with asterisks in Figure 9b), what is the likely reason for all sequences being merged in RC254. In Figure 9c it is shown the merging of all sequences from MC248, divided in two main branches, with one sequence (gi7297676) from MC249 (size 3), closely placed between them. The two remaining sequences from MC249 have built a duplet with each other (represented as the dashed branch) and none of them included gi7297676 when they used as Seed. The last case of partial merging of clusters occurred for RC12 that merged all sequences from MC4 with four out of five sequences from MC2, while the remaining sequence from MC2, that was not included in RC12, has built the RC11 by recruiting two additional sequences. All these examples illustrate the consistence of clusters generated by Single Linkage that merge proteins judged as distantly related by manual curation.

### Comparison to MultiParanoid approach

We compared our approach to another method, MultiParanoid, that has been shown to be very efficient in defining inparalog and ortholog clusters among multiple proteomes [4]. The result of the comparison of Seed Linkage versus MultiParanoid using the manually curated clusters as reference is shown in Table 1. While MultiParanoid produces a slightly reduced number of clusters (214) as compared to the manual curation (221), Seed Linkage produced 263 RC. This might indicate that Seed Linkage is more stringent for propagating information between clustered sequences since Seed Linkage yielded a larger number of clusters (59 clusters are a subset of the manual ones and 15 MC are split into distinct RC). Furthermore, Seed Linkage has rebuilt clusters with a larger number of seed sequences, since the number of singlets is rather small (only 38 against 179) as compared to MultiParanoid. Addition of sequences not included in curation was not restricted to Seed Linkage given that MultiParanoid presented a compatible number. As mentioned above, 153 of them provide evidence of being appropriately gathered as judged by the criteria used during curation – detection of over two TM domains. Agreement with manual curation seems weak at first glance, since only 13 RC are a perfect match as compared to 132 MultiParanoid clusters; however, in 100 RC all additional sequences display over two TM domains, thus 113 RC compare better with these 132 MultiParanoid clusters.

**Table 1:** Comparison of Seed Linkage *versus *MultiParanoid using manually curated clusters as a reference.

	**Seed Linkage**	**MultiParanoid**
Number of RCs	263	214
Additional sequences	275 (153^a^)	224
Singlets (non clustered manual sequences)	38	179
RC = MC (perfect match)	13	132
RC = MC + additional sequences	118 (100^a^)	28
RC = MC - some manual sequences^b^	59	17
MC is split in two or more RC^b^	15	9
Two or more MC are merged in RC^b^	15	9

^a^additional sequences display two or more TM segments; ^b^including or not additional sequences, MC: manually curated clusters, RC: rebuilt clusters.

As mentioned above, 15 Seed Linkage RC merge MC, but this is not restricted to our procedure, since in nine events a MultiParanoid is split in two or more Manual [4].

### Usage with partial proteomes

**Figure 10:**
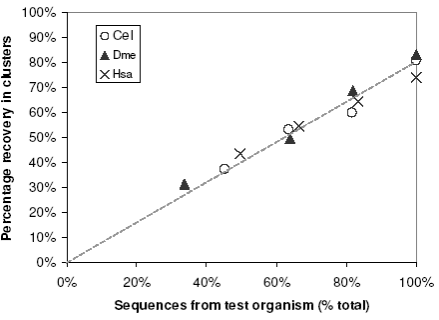
**Simulation of a novel and unfinished genome**. Seed Linkage rebuilt clusters containing four or more proteins from the indicated (test) organism were selected and 0 – 3 paralogs were artificially removed. Sequences of the other two complementary organisms were used as Seed and the percentage of recovery of the sequences from the indicated organism in clusters is shown. Complete recovery of paralogs would be represented by a diagonal line from zero up to 100% in both axes.

We selected from the 263 RC generated by Seed Linkage those clusters containing four or more sequences from organisms which we would artificially deplete to simulate an unfinished genome. For worm (Cel), fly (Dme) and human (Hsa), the respective set of 21, 15 and 88 clusters contained 115, 83 and 524 sequences. For each test, we randomly deleted 0 – 3 sequences from each of the 21 (Cel test), 15 (Dme test), or 88 (Hsa test) clusters, in turn, then dissolved the clusters. We then attempted to rebuild them using only the sequences of the remaining two organisms as Seeds (any remaining sequences from the test organism were present but not used as Seeds). The results are shown in Figure 10. With zero deletions from the test organism (X axis value at 100%), about 80% of the sequences were clustered by the Seeds from the other two organisms. This indicates that the clustering of the remaining 20% sequences from the test organism depends, at least in part, on paralogs from that test organism. Deletion of 1, 2, or 3 sequences per cluster yielded a linear recovery rate. For example, for fly, when three sequences per cluster were removed (retaining 34% of sequences in the recruitable set), 30% of all fly sequences (93% of the recruitable set) were clustered (triangles). The average clustering for each depletion test was 82% (that is, 82% of the clusterable sequences did indeed cluster) indicating that Seed Linkage can be applied to unfinished proteomes as well.

### Usage with a single iteration

Besides being used up to convergence, Seed Linkage can be used in single-run mode (r parameter = 0). A comparison of performance is shown in Table 2, together with an execution that limited the gathering of only one Reciprocal Best Hit (RBH) per Seed per organism (one inparalog in the Seed organism and one ortholog in Other Species). Clustering with the default usage produced clusters of mean size similar to the manual ones, while RBH grouped 1449 sequences in 402 clusters. The recall rate (sensitivity) that represents the amount of manual sequences that were clustered (not singlets) was comparatively high (97%) for both single iteration and convergence. Determination of Sensitivity with the raw results favors RBH execution; however, if one considers the recruited additional sequences that bear two or more trans-membrane domains (TM) as valid gathering events, then the Specificity* favors both single iteration and convergence. Thus, the advantage of using the convergence method seems to be increasing the linkage between clusters, resulting in a closer approximation of their number and mean size to the manual set.

**Table 2:** Comparison of Seed Linkage usage under different iterations.

	Manual	RBH	One iteration	Convergence
Sequences	1363	1449	1605	1638
Clusters	221	402	302	263
Mean cluster size	6.2	3.6	5.3	***6.2***
Singlets	0	158	44	38
Originals in clusters	1363	1205	1319	1325
Sensitivity^a^	100%	88%	***97%***	***97%***
Additionals	0	87	242	275
Additionals < 2 TM	0	6	28	36
Specificity^b^	100%	***93%***	84%	83%
Specificity*^,c^	100%	89%	***96%***	***96%***

^a^Sensitivity = Originals in clusters/Total of Manual Sequences; ^b^Specificity = True Positives/Total of Sequences; ^c^Specificity* was determined considering sequences with 2 or more TM domains as True Positives.

## Discussion

Seed Linkage was developed as an application to enrich the knowledge of similar proteins in species other than the Seed Organism. The present large size of proteome databases such as UniProt [12] suggests that a BLAST search involving all sequences against themselves would require an excessive amount of processing. Seed Linkage simplifies the search, focusing on the subjects of the Seed.

Similar to the approach used by Inparanoid, particular importance is given to the score between Seed and the best scoring Seed subject that establishes with Seed a Bidirectional Best Hit (BBH) relationship. Using a manually-curated dataset of trans-membrane proteins from worm, fly and man, we found that 37% of all gathering events (Figure 6a) were initiated by finding a BBH subject (BBHsj) in a species different from the Seed Organism. When this happens, the *inparalog retrieval score limit *is made equal to the score obtained by the alignment of Seed and BBHsj, both within the Seed Organism and in the Candidate Species as well (Figure 1). Most of the grouped sequences when a BBHsj is attained corresponded to the ones listed in the manually curated database used as reference. However, it is possible that additional sequences are actually correct recruitments (Figure 8 and Table 1).

Seed linkage then continues the search for related proteins by two means, aiming to group inparalogs both in Seed Organism, using Seed as bait, and in Candidate Species, using the BBH relationship established by a recruited inparalog in the Seed Organism. We tested the performance of Seed Linkage and found better results when the iterative searches of sequences in Candidate Species that are initiated by a recruited inparalog is limited by the initial score between Seed and its BBHsj (data not shown), which makes the procedure more robust since one intended use of Seed Linkage is to identify circumstances that warrant propagation of information associated with Seed.

Inparalogs from the Seed Organism often are able to simultaneously recruit additional inparalogs in the Seed Organism, by establishing an additional BBH relationship with a Candidate Species. For example, from all gathering events in a cluster initiated by a Seed-BBHsj match, 11% do not involve the Seed itself.

Special attention was given to cases when a BBHsj was not found, to establish an *inparalog retrieval score limit*. For the studied curated database, such events occurred often (58%, Figure 5a, adding the last two bars). Two possible limits were investigated: a raw score and a relative score. The relative score was chosen to reflect a proportion of the score that the Seed shows when aligned to itself, thus incorporating the information of its size. Analysis of the distribution of the scores during the event of recruitment allowed us to find an empirical value for the *inparalog retrieval score limit*, so we could attain results that are similar to the processes initiated in the presence of a BBH relationship between Seed and BBHsj. The greatest performance was yielded by setting the value to 0.3 (Figure 5b), which reduced the recruitment of additional sequences to an acceptable rate. In our implementation, we provide support for users who wish to apply more stringent limits based on inspection of Figure 5. In the absence of a BBHsj, the *inparalog retrieval score limit *of 0.3 might benefit of an adjustment *a posteriori *to adapt to each protein family. Further studies in this respect are envisaged.

Many databases group similar proteins and propagate the information amongst the members. Two examples of such databases are GOA (Gene Ontology Annotation, by EBI [13]) and KOG (the eukaryotic version of COG, by NCBI [6]). If we consider a database as a table composed of a column for each organism and a row for each protein, it might be noticed that GOA prioritizes the enlargement of the columns, which will contain very different number of gene entries per organism. Conversely, databases such as KOG strictly target the completion of the rows, listing all genes with similar function in the constituting organisms. Several approaches tend to focus more on these rows (proteins) than to enlarge the columns (organisms), although they might actually work on between those goals, such as Inparanoid/Multiparanoid, OrthoMCL [14], Kegg Orthology [15], and EGO [16], amongst others. Seed Linkage joins these efforts with a declared option for grouping cognate proteins from multiple organisms beginning with only one sequence, through connectivity saturation with that Seed sequence. As an example, using a protein from a dicot plant as Seed, it might be able to gather similar proteins in monocot plants, which in turn can act as a better reference sequence for similarity searches in the monocot Species. Moreover, Seed Linkage adds two relevant functionalities: (i) it does not require the Candidate Species to have completed genome and (ii) it saves computing time since it does not require alignment of all sequences to each other.

Certainly the recruitment yielded by Seed Linkage is amenable to additional approaches to validate the clustering such as literature support [17], mapping of conserved domains [18], alignment of secondary structure, etc. Indeed, a comparative analysis of Seed Linkage and Multiparanoid did not yield the same results (Table 1), although a similar number of clusters and performance were obtained. However, Seed Linkage recruits, with an acceptable level of confidence, a significant number of candidates, maximizing the search on all available proteomes while minimizing computing time. The software is made available for the research community, and a web service dedicated to Seed Linkage is currently under construction. Seed Linkage is also currently being used to construct a Database for Protein Defense Mechanisms in plants.

## Conclusion

The Seed Linkage software was produced with the aim of clustering cognate proteins from multiple organisms beginning with a single sequence through connectivity saturation with that Seed sequence. The method results were comparable to conventional clustering approaches. Generating clusters based only on a protein of interest is less time consuming than generating clusters for whole proteomes, and can be applied to establish members from unfinished proteomes as well.

## Availability and requirements

• **Project name: **Seed Linkage clustering of related protein sequences;

• **Project home page: **;

• **Operating system: **Linux;

• **Programming language: **PHP;

• **Other requirements: **PHP 5 or higher, MySQL 5 or higher, NCBI BLAST package;

• **Any restrictions to use by non-academics: **License needed.

• **NCBI Taxonomy database:**

## Authors' contributions

AB-S created, implemented and tested the proposed algorithm; created and conducted the pilot tests and wrote the paper. VPS helped in the algorithm discussion, development and implementation. RS and JMO advised in the algorithm implementation, discussed the pilot tests and coordinated the method development. JMO created the paper's main idea and supervised the writing of the paper. All authors read and approved the final manuscript.

## Supplementary Material

Additional file 1 Seed Linkage Package. Compressed core of scripts written in PHP necessary to run the Seed Linkage procedure. Also contains a documentation file about each sub item. Click here for file
